# Supplementary material for: Structural Analysis of Plasmodium falciparum Hexokinase Provides Novel Information about Catalysis Due to a Plasmodium-Specific Insertion
Source: Int J Mol Sci. 2023 Aug 13;24(16):12739. doi: 10.3390/ijms241612739 (PMC10454665; doi:10.3390/ijms241612739)
Supplement: Supplementary file 1 [file ijms-24-12739-s001.zip › ijms-2553114-supplementary.pdf]

Supplemental Information

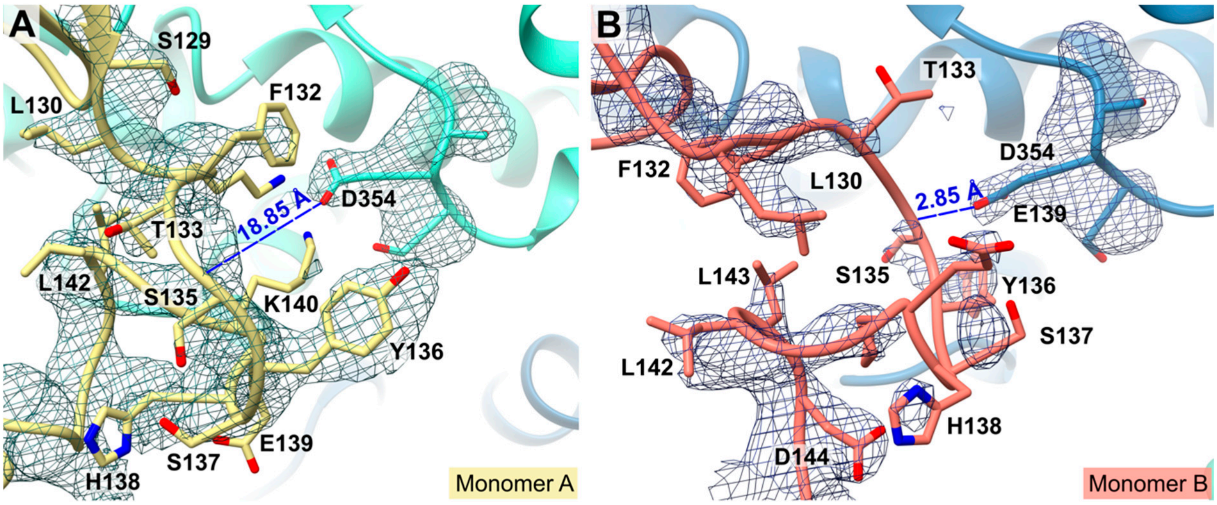

Figure S1. Electron density of the *Plasmodium*-specific insertion (P-insert) and structural comparison with *Eimeria tenella* hexokinase (*Et*HK).
(A) Close-up of the P-insert from monomer A. The final omit map covers the P-insert and residues S353-S355 and is contoured at 2.5 σ. The distance between S135 and D354 is 18.85 Å. **(B)** Close-up of the P-insert from monomer B. The final omit map covers the P-insert and residues S353-S355 and is contoured at 2.4 σ. The distance between residue S135 (N) and D354(OD2) is 2.85 Å.

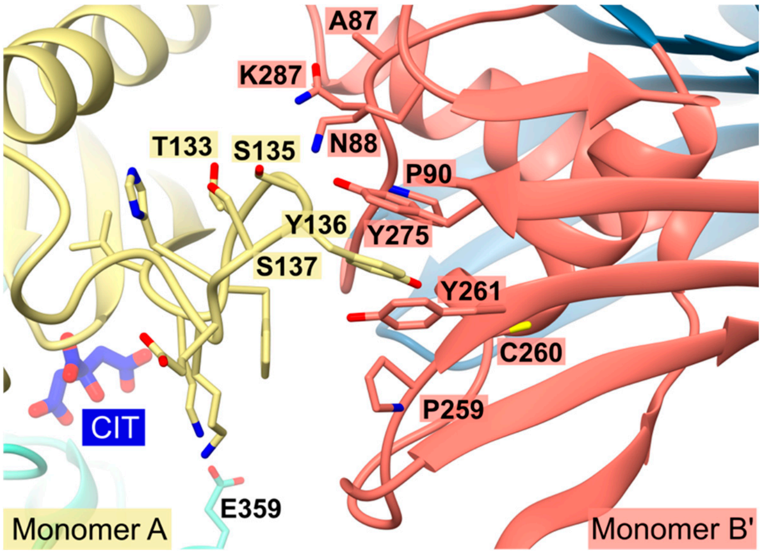

Figure S2. P-insert residues interact via hydrophobic interactions with the neighboring tetramer. Close-up of the P-insert from monomer A. Residues G134-Y136 interact with residues N87-P90, Y275, K287, and C260-Y261 of subunit B'.
CIT, citrate

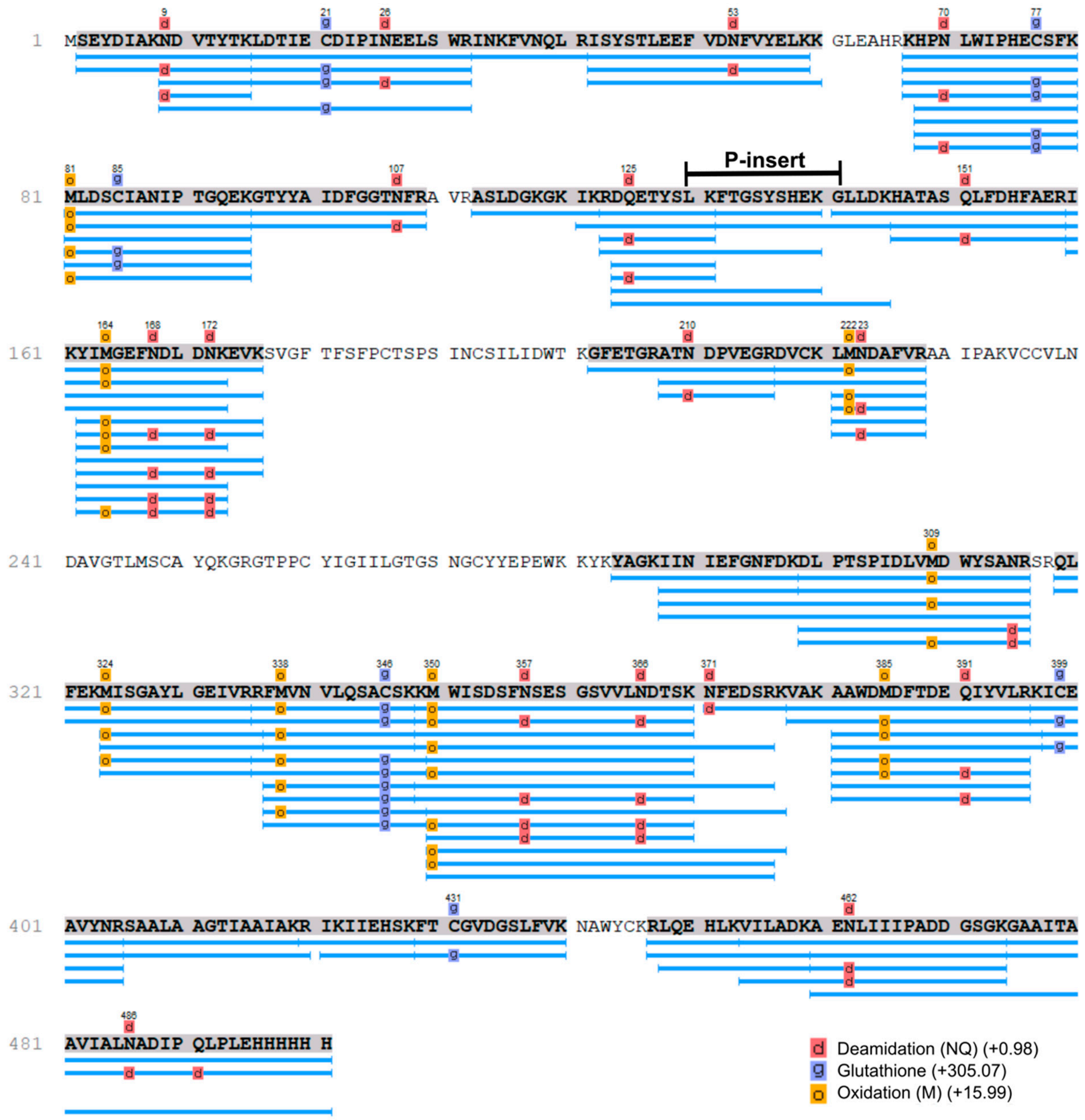

Figure S3. Protein coverage of S-glutathionylated *Pf*HK after mass spectrometry analysis. Modified protein samples were analyzed with the software PEAKS. Peptides found in mass spectrometry analysis are shown in blue lines under the respective amino acids (one-letter code). S-glutathionylated cysteines are indicated with blue squares (C21, C77, C85, C346, C399, C431) and were detected by a shift of mass (+305.07 Da).

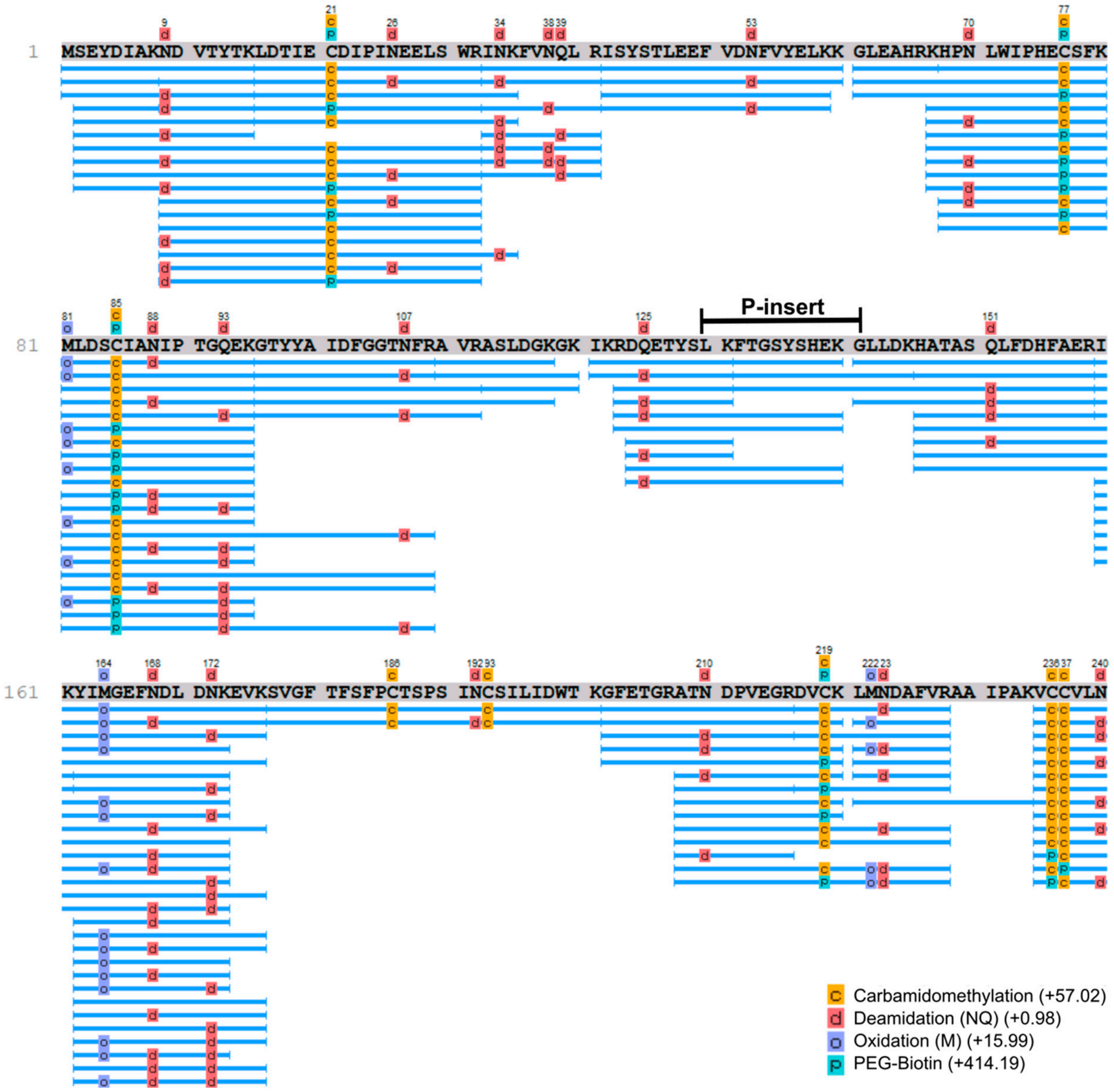

Figure S4 (a). Protein coverage of S-nitrosated *Pf*HK after mass spectrometry analysis.

Modified protein samples were analyzed with the software PEAKS. Peptides found in mass spectrometry analysis are shown in blue lines under the respective amino acids (one-letter code). S-nitrosated (biotinylated for the analysis) cysteines are indicated with green squares (C21, C77, C85, C219, C236, C237, C249, C260, C273, C346, C399, C431) and were detected via a shift of mass (+414.19 Da).

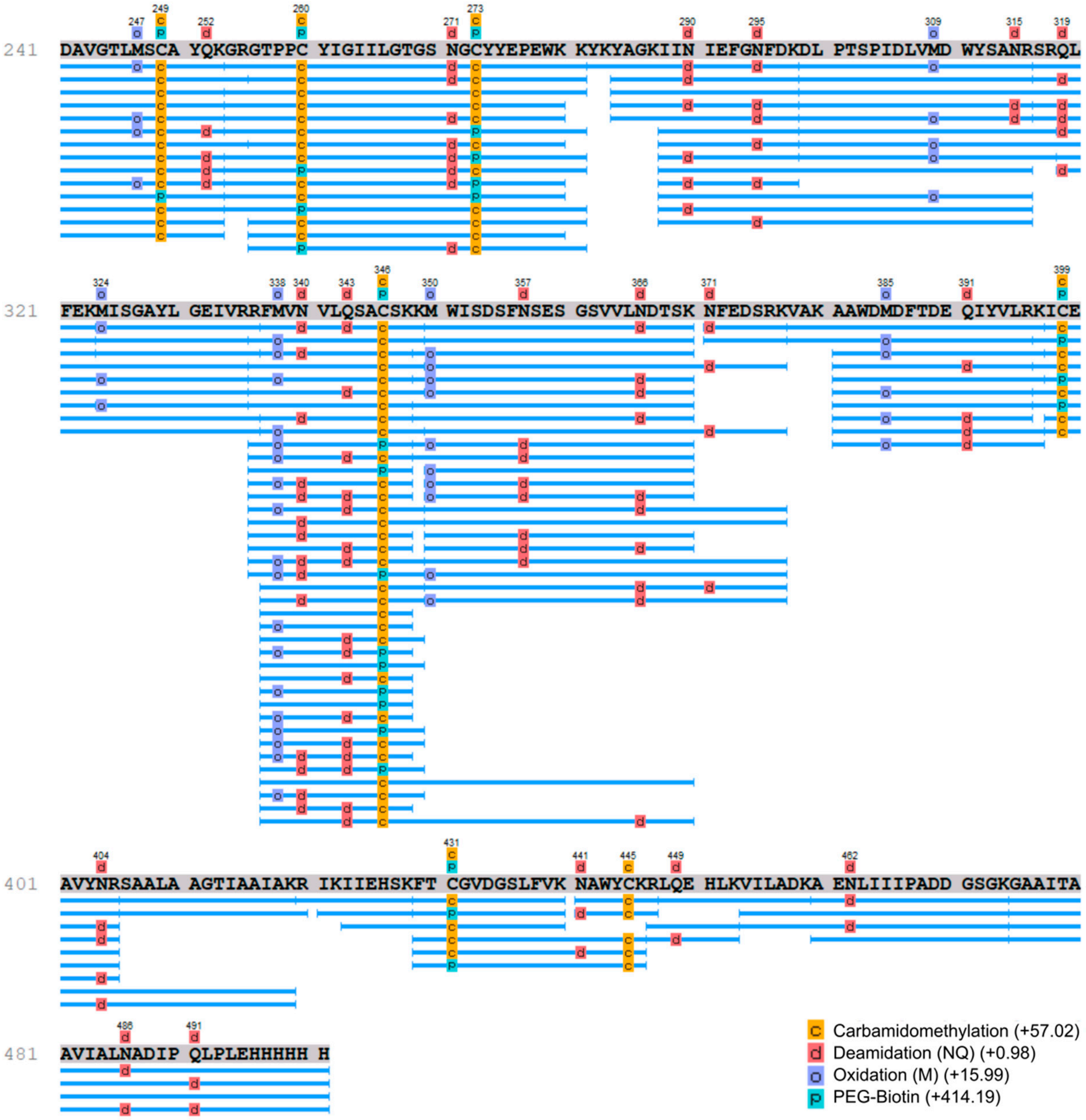

Figure S4 (b). Protein coverage of S-nitrosated *Pf*HK after mass spectrometry analysis.

Modified protein samples were analyzed with the software PEAKS. Peptides found in mass spectrometry analysis are shown in blue lines under the respective amino acids (one-letter code). S-nitrosated (biotinylated for the analysis) cysteines are indicated with green squares (C21, C77, C85, C219, C236, C237, C249, C260, C273, C346, C399, C431) and were detected via a shift of mass (+414.19 Da).

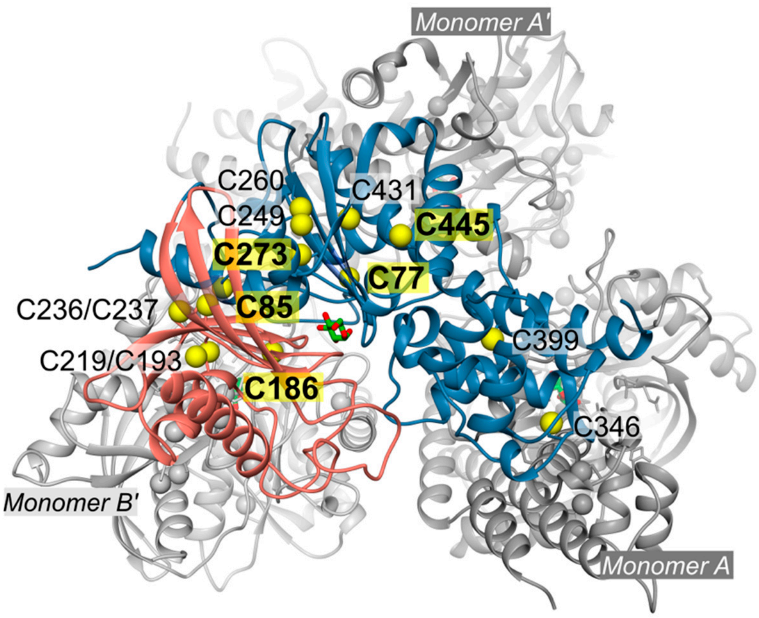

Figure S5. Distribution of cysteines in *Pf*HK monomer B.

The *Pf*HK monomer contains 15 cysteines, but only a few are probably involved in regulating the enzymatic activity via oxPTMs. No electron density could be detected for C21. C77 and C186 are located near the active site, C193 and C346 are part of the tetrameric interface. The active site is marked by GLC, coloured in green.

```
Pgallinaceum       300   LPLAPIDLVMDWYSSNRSRQLFEKMISGAYLGEMVRRYMVNVLQSASSKKMWITDTFNSE   359
Prelictum          300   LPLAPIDLVLDWYSSNRSRQLFEKMISGAYLGEIVRRYMVNVLQSSSSKKMWISDTFNSE   359
Padleri            300   LPTSPIDLVMDWYSANRSRQLFEKMISGAYLGEIVRRFMVNVLQSACSKKMWISDSFNSE   359
Pgaboni            300   LPTSPIDLVMDWYSANRSRQLFEKMISGAYLGEIVRRFMVNVLQSACSKKMWISDSFNSE   359
Pbillcollinsi      300   LPTSPIDLVMDWYSANRSRQLFEKMISGAYLGEIVRRFMVNVLQSACSKKMWISDSFNSE   359
Pblacklocki        300   LPTSPIDLVMDWYSANRSRQLFEKMISGAYLGEIVRRFMVNVLQSACSKKMWISDSFNSE   359
Ppraefalciparum    300   LPTSPIDLVMDWYSANRSRQLFEKMISGAYLGEIVRRFMVNVLQSACSKKMWISDSFNSE   359
Preichenowi        300   LPTSPIDLVMDWYSANRSRQLFEKMISGAYLGEIVRRFMVNVLQSACSKKMWISDSFNSE   359
Pfalciparum        300   LPTSPIDLVMDWYSANRSRQLFEKMISGAYLGEIVRRFMVNVLQSACSKKMWISDSFNSE   359
Pmalariae          300   LPLSPIDLVMDWYSANRSRQLFEKMISGAYLGEIVRRFMVNVLQSDSSSKMWNNDSFNSE   359
Povalecurtisi      300   LPLSPIDLVMDWYSANRSRQLFEKMISGAYLGEIVRRFMVNVLQSASSKKMWKSDSFNSE   359
Pvivax             300   LPLSPIDLVMDWHSANRSRQLFEKMISGAYLGEIVRRFMVNVLQSASSEKMWKSDSFNSE   359
Pknowlesi          300   LPLTPIDLVMDWYSANRSRQLFEKMISGAYLGEIVRRFMVNVLQSASSEKMWKSDSFNSE   359
Pvinckei           300   LPLSPVDLVMDWYSANRSRQLFEKMISGAYLGEIVRRFMVNVLQSESSKKMWQSDSFSSE   359
Pchabaudi          301   LPLSPIDLVMDWYSANRSRQLFEKMISGAYLGEIVRRFMVNVLQSASSKKMWQSDSFNSE   360
Pyoelii            301   LPLSPIDLVMDWYSANRSRQLFEKMISGAYLGEIVRRFMVNVLQSASSKKMWQSDSFNSE   360
Pberghei           301   LPLSPIDLVMDWYSANRSRQLFEKMISGAYLGEIVRRFMVNVLQSASSKKMWQSDSFNSE   360
Pinui              300   LPLSPVDLVMDWYSANRSRQLLEKMISGAYLGEIVRRFMVNVLQSASSEKMWKSDSFDSE   359
Pcynomolgi         300   LPLSPIDLVMDWHSANRSRQLFEKMISGAYLGEIVRRFMVNVLQSESSEKMWKSDSFNSE   359
Pvivaxlikesp       300   LPLSPIDLVMDWHSANRSRQLFEKMISGAYLGEIVRRFMVNVLQSASSEKMWKSDSFNSE   359
Pfragile           300   LPLTPIDLVMDWYSANRSRQLFEKMISGAYLGEIVRRFMVNVLQSASSEKMWKSDSFNSE   359
                         ** :*:***:**:*:******:***********:***:******* .*.*** .*:*.**
```

Figure S6. Multiple sequence alignment of different *Plasmodium* species.

C346 is conserved among *P. falciparum* and six other *Plasmodium* species pathogenic to chimpanzees and gorillas. Sequences were analyzed using Clustal Omega Multiple Sequence Alignment.
